# From cell atlases to mechanisms: bridging scRNA-seq discovery with in vivo genetics

**DOI:** 10.1038/s44318-026-00846-5

**Published:** 2026-07-03

**Authors:** Bin Zhou

**Affiliations:** 1 CAS CEMCS-CUHK Joint Laboratory, New Cornerstone Science Laboratory, State Key Laboratory of Cell Biology, Shanghai Institute of Biochemistry and Cell Biology, Center for Excellence in Molecular Cell Science, Chinese Academy of Sciences, Shanghai, China; 2 CAS CEMCS-CUHK Joint Laboratory, Department of Chemical Pathology, The Chinese University of Hong Kong, Prince of Wales Hospital, Hong Kong, China; 3 Key Laboratory of Systems Health Science of Zhejiang Province, School of Life Science, Hangzhou Institute for Advanced Study, University of Chinese Academy of Sciences, Hangzhou, China; 4 School of Life Science and Technology, ShanghaiTech University, Shanghai, China

**Keywords:** Chromatin, Transcription & Genomics, Genetics, Gene Therapy & Genetic Disease, Methods & Resources

## Abstract

By the intersection of two markers, SIRA provides a practical route from single-cell atlas data to causal in vivo functional interrogation of cellular subpopulations.

**Figure:**
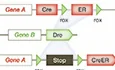

## Teaser

Single-cell RNA sequencing has uncovered vast cellular heterogeneity, yet the in vivo functions of most newly identified subpopulations remain unclear, risking an “atlasing valley of death”, in which descriptive catalogs remain untethered to mechanism. To address this gap, I propose a genetic Sequential Intersectional Recombinase Approach (SIRA) that pairs a cell-type-specific Dre driver with a subpopulation-enriched gene-driven rox-Stop-rox-CreER allele. SIRA restricts Cre activity to cells at the intersection of two genetic markers, allowing precise lineage tracing, targeted ablation, fate manipulation, and floxed-allele perturbation in defined subpopulations. By operationalizing intersectional genetics downstream of discovery-based omics, SIRA provides a practical path from atlas to mechanism in vivo.

A fundamental goal of modern biology is to understand how cells execute their functions in vivo. Gene expression profiles can suggest potential, but they do not reveal actual behavior—whether a cell proliferates, migrates, differentiates, or drives disease. The disconnect arises because molecular abundance does not equate to functional output. Achieving mechanistic insight requires moving from correlation to causation, from descriptive gene lists to direct experimentation in living organisms. Yet many studies end at molecular profiling, leaving the functions of newly identified cell populations unresolved. This gap between what cells express and what they do is a major bottleneck in translating discovery into biological understanding and application. Bridging this divide is essential for understanding development, maintaining homeostasis, and treating disease.

## The era of single-cell atlases for cataloging cellular heterogeneity

Single-cell RNA sequencing (scRNA-seq) has revolutionized our ability to catalog cellular heterogeneity. Since its first description in 2009 (Tang et al, 2009) and the advent of widely accessible commercial platforms by 2015, most notably the 10x Genomics Chromium (Zheng et al, 2017), scRNA-seq has accelerated discoveries across developmental biology, cancer, immunology, and regenerative medicine. Ongoing advances in barcoding, multiplexing, and computational analysis have transformed scRNA-seq into a standard tool, enabling comprehensive efforts such as the Human Cell Atlas (Regev et al, 2017). The field continues to expand rapidly into nearly every domain of life science and medicine. A key outcome of this technological revolution is the discovery of previously unrecognized cellular subpopulations in virtually every tissue and organ system. Defined by distinct marker combinations, transcriptional states, or transitional trajectories, these subpopulations have been uncovered in contexts ranging from early embryogenesis to tumor microenvironments, from fibrotic hearts to inflamed intestines. Some represent rare or transient states invisible to bulk profiling; others reveal unexpected heterogeneity within seemingly uniform cell types. Tens of thousands of studies now report such subpopulations, each with ranked signature genes and predicted functions. In many cases, however, this descriptive census has become the endpoint—an atlas in search of mechanistic meaning.

## The disconnect from description to mechanism

Cataloging heterogeneity is not the same as understanding it. A growing gap separates the cells we can now name from the functions we can confidently assign to them in vivo. Many scRNA-seq studies end with descriptive output such as subpopulation calls, ranked marker genes, and pathway enrichments, while leaving in vivo roles largely unexplored. Where are these cells located, and what are their morphologies? What is the consequence of ablating them, or of disrupting their signature genes in disease? How are their fate and functions regulated across disease stages? Transcriptional profiles alone cannot answer these questions. As Peter Carmeliet and colleagues have emphasized, the field is awash in ranked gene lists and putative functions, but rigorous in vivo validation remains scarce (Sokol et al, 2023). Genomics has excelled at cataloging; the next challenge is causality. Historically, decisive advances have come from perturbation—from Morgan’s Drosophila genetics (Morgan, 1910) to Capecchi’s gene targeting in mice (Thomas and Capecchi, 1987)—not from correlation. Without systemic functional tests, single-cell omics risks an “atlasing valley of death”: ever-expanding catalogs of cellular heterogeneity untethered from physiological or pathological context.

## The limitation of single-marker Cre drivers

**Figure 1:**
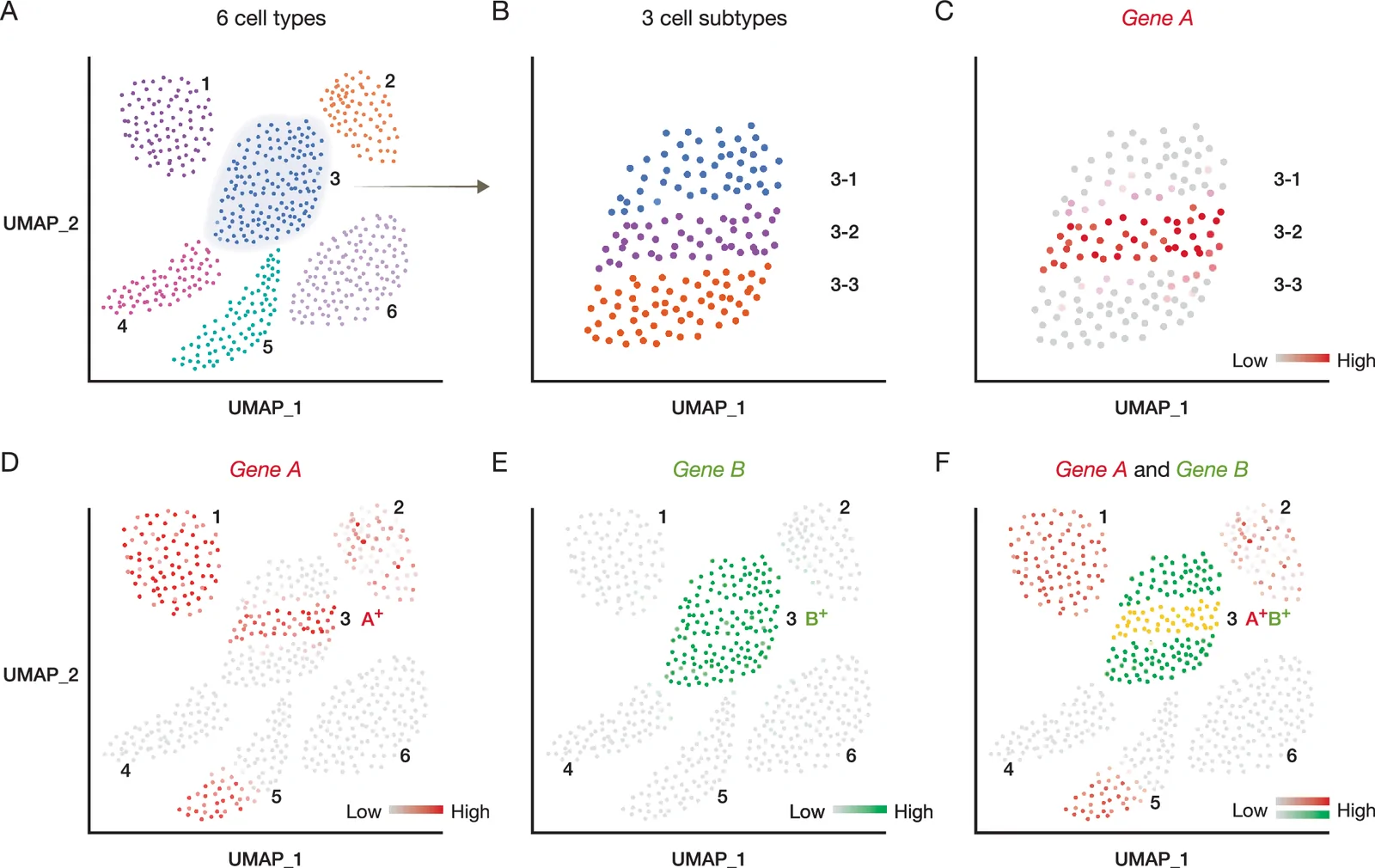
Subpopulations defined by two markers. (**A**) UMAP visualization of annotated major cell types. Cells are colored according to their respective annotations. (**B**) UMAP visualization showing the subclustering of cell type 3 (e.g., fibroblasts) into three distinct subpopulations (3-1, 3-2, and 3-3). (**C**) Feature plot showing expression of *Gene A* within fibroblast subpopulations identified in Figure B. The color represents the expression level. (**D**) Feature plot showing the expression of *Gene A* across all cell populations. (**E**) Feature plot showing the expression level of *Gene B* across all cell populations. (**F**) Feature plot showing co-expression of *Gene A* and *Gene B* across all cell populations; the target subpopulation is defined as B⁺ and A⁺.

The Cre-loxP system remains the gold standard for in vivo functional genetics (Sauer and Henderson, 1988), enabling cell-type-specific knockout, activation, ablation, fate manipulation, and lineage tracing, especially when paired with inducible variants such as Cre-ERT2 (Feil et al, 1997). A critical limitation arises, however, when probing *subpopulations* within a major cell type. Consider a typical scRNA-seq workflow: unsupervised analysis reveals six major cell types, visualized on Uniform Manifold Approximation and Projection (UMAP) or t-distributed Stochastic Neighbor Embedding (t-SNE) embeddings (Fig. 1A). Within one such as fibroblasts (cell type 3), subclustering reveals three subpopulations (3-1, 3-2, 3-3) (Fig. 1B). Subpopulation 3-2 is flagged for a putative role in inflammatory fibrosis based on Gene Ontology enrichment of its upregulated genes, and *Gene A* is among the top differentially expressed markers distinguishing 3-2 from 3-1 and 3-3 (Fig. 1C). The intuitive next step is to generate an *A-CreER* mouse line to lineage-trace and manipulate *Gene A*⁺ fibroblasts. The problem is that “specificity” judged within a single lineage often collapses when viewed across tissues. In a pan-tissue context, *Gene A* also marks cells in other lineages—robustly in cell type 1, sparsely in cell type 2, and within a subpopulation of cell type 5 (Fig. 1D). Thus, an *A-CreER* driver alone would label *Gene A*⁺ cells across multiple lineages, confounding interpretation of 3-2 fibroblast-specific functions. True exclusive markers— genes expressed *only* in the target subpopulation and nowhere else—are exceptionally rare. As a result, many so-called “cell type-specific” Cre lines in practice drive recombination in broader populations than their names imply. The challenge is even greater if the marker is pleiotropically expressed in different cell types across organs, limiting organ-axis studies (e.g., heart–brain or gut–liver interactions).

## SIRA: a sequential intersectional recombinase approach

How can we gain genetic access to a subpopulation defined not by a single marker but by the combinatorial logic of two? The solution is intersectional genetics. If *Gene B* is a bona fide pan-fibroblast marker (e.g., *Col1a2*, *Col1a1*, or *Pdgfra*) and *Gene A* is enriched in the 3-2 fibroblast subpopulation, then the target is the B⁺A⁺ intersection (Fig. 1F). Orthogonal dual-recombinase systems—such as Flp-Cre (Awatramani et al, 2003), Dre-Cre (He et al, 2017b), or Nigri-Cre (Liu et al, 2018)—can, in principle, specify such intersections with high precision. However, previous strategies largely drive dual-logic reporters (e.g., AND-gate activation from *Rosa26*) and cannot leverage the vast repertoire of existing Cre-responsive loxP-floxed alleles, which are not substrates for other recombinases like Dre or Flp. This limitation is especially constraining for functional studies.

**Figure 2:**
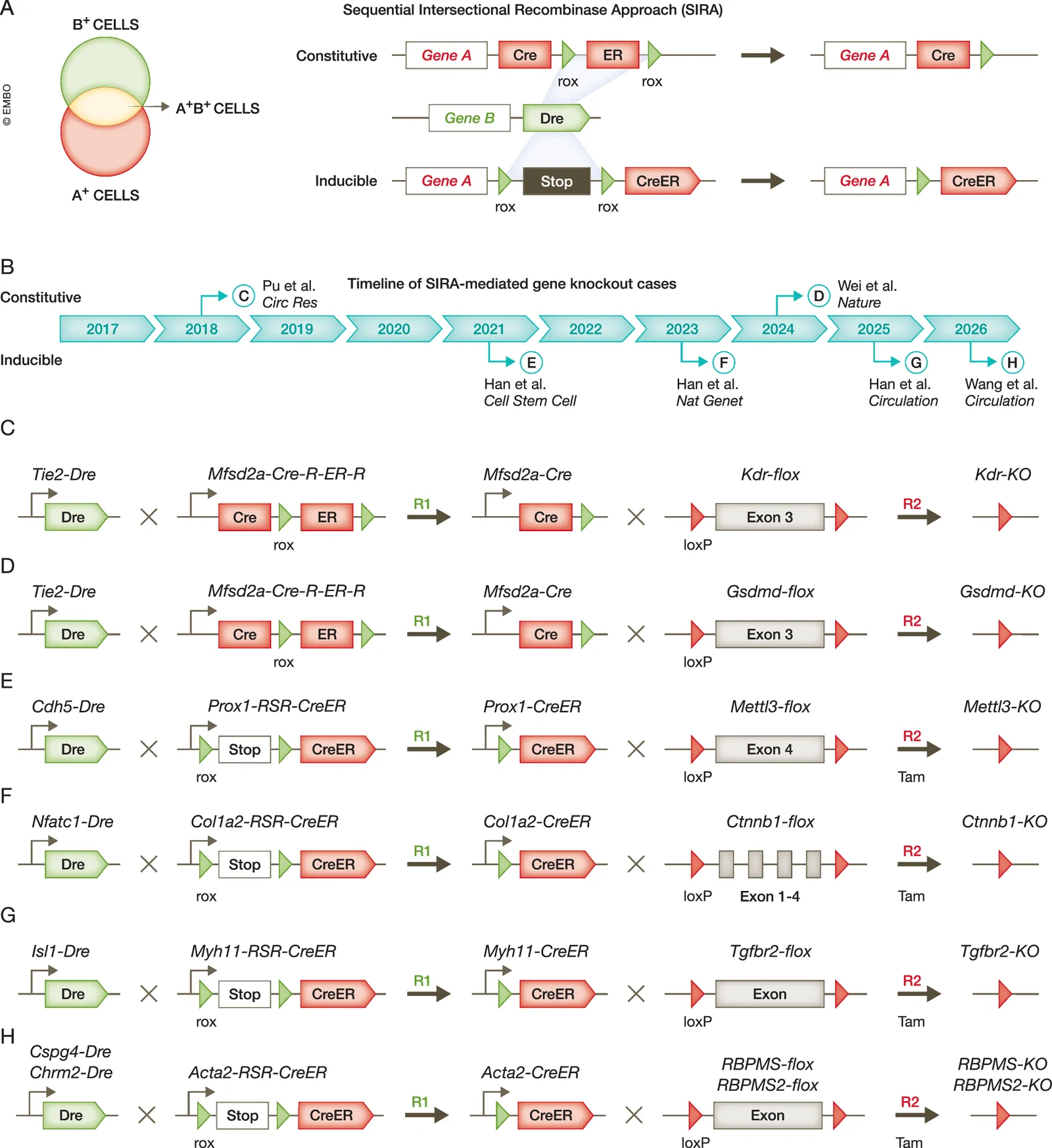
SIRA enables precise genetic targeting of subpopulations. (**A**) Schematic of the sequential intersectional recombinase approach (SIRA) for targeting cells expressing both *Gene A* and *Gene B* using a constitutively active Cre or inducible CreER strategy. (**B**) Schematic showing the timeline of mediated gene knockout cases. (**C**, **D**) Schematic showing SIR for conditional knockout Kdr (**C**) or Gsdmd (**D**) in BBB using *Tie2-Dre* and *Mfsd2a-Cre-R-ER-R* mice. (**E**) Schematic showing SIRA for conditional gene deletion in lymphatic endothelial cells using *Cdh5-Dre* and *Prox1-RSR-CreER*. (**F**) Schematic showing SIRA for conditional gene deletion in endocardium-derived fibroblasts using *Nfatc1*-*Dre* and *Col1a2*-*RSR-CreER*. (**G**, **H**) Schematic showing SIRA for conditional gene knockout in different subpopulations of smooth muscle cells. R1, Dre-rox recombination; R2, Cre-loxP recombination.

To overcome this limitation and unlock access to the entire Cre-loxP toolkits, we propose a Sequential Intersectional Recombinase Approach (SIRA). Using Dre-Cre as a proof of principle, a *B-Dre* driver marks all B⁺ cells (the parent lineage). In a parallel configuration, an *A-Cre-R-ER-R* construct fuses Cre with a rox-flanked ER cassette, resulting in cytoplasmic retention of the Cre-R-ER-R fusion protein. Upon Dre-mediated excision of the ER cassette, Cre becomes free to translocate to the nucleus and drives recombination, effectively operating as a constitutive Cre strategy (Fig. 2A). Alternatively, an *A-RSR-CreER* knock-in places CreER downstream of a rox-Stop-rox (RSR) cassette, rendering CreER expression inactive until Dre excises the transcriptional Stop element (Fig. 2A). In this inducible strategy, Dre-mediated rox recombination within B⁺ lineage unlocks A-driven CreER expression. As a result, CreER becomes active exclusively in the B⁺A⁺ intersection – the 3-2 subpopulation (Fig. 2A). SIRA enables lineage tracing, targeted ablation, fate manipulation, and precise perturbation of any loxP-floxed allele. In doing so, SIRA operationalizes intersectional logic while retaining full compatibility with existing Cre-loxP resources, providing a practical bridge from discovery-omics to hypothesis-driven in vivo genetics.

## Validating SIRA for in vivo floxed gene deletion

We validated SIRA for subpopulation-specific gene manipulation in four examples (Fig. 2B). The first example illustrates the constitutive Cre strategy following Dre-rox recombination (Fig. 2C). To target brain endothelial cells (BECs) for investigating their role in the blood-brain barrier (BBB), we leveraged the intersection of *Tie2* and *Mfsd2a* expression: *Tie2* marks endothelial cells across all organs, whereas *Mfsd2a* is expressed in both BECs and neurons. We combine *Tie2-Dre* driver with an *Mfsd2a-Cre-R-ER-R* line. Dre-mediated excision of rox-flanked ER cassette yielded *Mfsd2a-Cre* activity restricted exclusively to BECs (Pu et al, 2018). Crossing this BEC-specific Cre line with *Kdr-flox* mice enabled targeted deletion of VEGFR2 in BECs, revealing its essential role in maintaining BBB integrity. In a subsequent application, we used the same BEC-Cre to specifically knock out *Gsdmd* in BECs (Fig. 2D). This work demonstrated that lipopolysaccharide-induced activation of the pore-forming protein GSDMD in BECs mediates BBB disruption (Wei et al, 2024).

Second, to target lymphatic endothelial cells (LECs), a molecularly and functionally distinct subset of endothelial cells, we combined a pan-endothelial *Cdh5-Dre* driver with a *Prox1-RSR-CreER* allele (Fig. 2E). Prox1, a master regulator of LEC fate (Wigle et al, 1999), is also expressed in neural stem cells, cardiomyocytes, hepatocytes, and epithelial cells (Han et al, 2021); thus, conventional *Prox1-CreER* manipulates multiple lineages and confounds interpretation (Srinivasan et al, 2007). By restricting CreER activation to the Cdh5⁺ lineage, SIRA confines recombination to the intersection of Cdh5 lineage and Prox1 expression, i.e., LECs. Crossing these mice to *Mettl3-flox* achieved efficient, LEC-specific deletion and revealed an essential role for Mettl3 in embryonic lymphatic vessel growth (Han et al, 2021). This established SIRA as a practical route to subpopulation-specific loss-of-function.

Third, to dissect endocardium-derived fibroblasts (EndoFb) in the heart, a subpopulation distinct from epicardium-derived fibroblasts (Moore-Morris et al, 2014), we paired *Nfatc1-Dre* (endocardium lineage) with *Col1a2-RSR-CreER* (fibroblasts) (Fig. 2F). Because the endocardium also contributes to coronary endothelial cells, smooth muscle cells (SMCs), and pericytes in the developing heart (Tian et al, 2014; Chen et al, 2016), *Nfatc1-Cre* alone is not EndoFb-specific; likewise, *Col1a2-CreER* labels epicardium-derived fibroblasts and fibroblasts in other organs (He et al, 2017a; Wu et al, 2021). Their intersection, however, precisely defines EndoFb (Fig. 2F). Guided by gene-set enrichment indicating elevated canonical Wnt signaling and proliferation in EndoFb, we deleted *Ctnnb1* (encoding β-catenin) specifically in this subpopulation and demonstrated that Wnt signaling drives EndoFb expansion during cardiac fibrosis (Han et al, 2023).

Fourth, SMCs are heterogeneous in terms of their developmental origins, gene expression, and function. Notably, SMCs of the ascending aorta, aortic arch, and descending aorta arise from distinct developmental origins. Han et al combined *Isl1-Dre* with *Myh11-RSR-CreER* to delete Tgfbr2 specifically in SMCs of the ascending aorta (Fig. 2G), resulting in aneurysm formation restricted to this region (Han et al, 2025). In addition, pairing *Wnt1-Dre* with *Myh11-RSR-CreER* enabled efficient Smad4 deletion in arch SMCs, demonstrating that Smad4 is essential for preserving SMC integrity in the aortic arch (Han et al, 2025). Conventional SMC-specific Cre lines suffer from off-target activity and cannot distinguish between arterial SMCs (ASMCs) and nonvascular SMCs (NVSMCs), limiting compartment-specific gene targeting and mechanistic insight. A recent study addressed this by developing *Cspg4-Dre* and *Chrm2-Dre*, each paired with *Acta2-RSR-CreER*, to specifically target ASMCs and NVSMCs, respectively (Fig. 2H). Global deletion of the splicing regulators RBPMS and RBPMS2 across SMCs abolished the contractile program in intestinal NVSMCs, resulting in fatal intestinal obstruction and early lethality (Wang et al, 2026). In contrast, ASMC-restricted Rbpms/2 deletion increased the thickness of the muscular layer in pulmonary arteries without causing any intestinal abnormalities (Wang et al, 2026).

Together, these examples illustrate that SIRA enables temporally controlled, subpopulation-restricted manipulation of any loxP-floxed allele while avoiding off-target recombination inherent to single-marker drivers. In principle, the strategy generalizes to any cellular subpopulation for which two orthogonal markers can be defined, and alternative recombinase pairs, such as Flp-Cre or Nigri-Cre, can be used to establish SIRA in vivo (Schonhuber et al, 2014; Liu et al, 2018).

## SIRA for dynamic and pathological cell states

Single-cell RNA sequencing increasingly reveals transient cell states that arise briefly during development, homeostasis, or disease. These states—often marked by hybrid transcriptional programs, metabolic shifts, or poised epigenetic landscapes—appear across contexts from embryonic morphogenesis to cancer metastasis and tissue fibrosis. Beyond stable subpopulations defined by static markers, SIRA offers a way to interrogate these short-lived, pathology-induced states. Many diseases—including chronic inflammation, fibrosis, atherosclerosis, and cancer—feature plastic, transient cell states that are rare or absent in health. These may represent reactive, proliferative, or de-differentiated cells that drive initiation, progression, or resolution. Because they are specified by dynamic gene expression programs rather than fixed lineage markers, such states have been difficult to study with conventional genetics. SIRA can be adapted to target these states by combining a lineage gate with a state-dependent gate. Concretely, a cell type-specific driver (e.g., B-Dre) restricts recombination to the parent lineage, while a disease-induced promoter/enhancer controls an *A-RSR-CreER* cassette that is activated only after Dre excises the Stop. Tamoxifen-timed CreER then confines labeling or gene manipulation to cells that enter the transient program within a defined temporal window. This enables (i) permanent lineage tracing of state-entered cells even after they revert, (ii) ablation or fate manipulation during the window of plasticity, and (iii) floxed-allele perturbation to test causal roles in vivo. The same logic is implementable with other orthogonal pairs, such as Flp-Cre and Nigri-Cre (Schonhuber et al, 2014; Liu et al, 2018) to study transient, high-plasticity states in diverse diseases, including lung cancer (Chan et al, 2026). Together, these adaptations pave the way to mechanistic dissection and potential therapeutic targeting of disease-driven cellular plasticity.

## A community-driven strategy for implementation

The era of single-cell discovery has delivered an extraordinary map of cellular diversity; the next era must be one of perturbation. SIRA provides a practical bridge from descriptive atlasing to mechanistic in vivo genetics. By converting any subpopulation-enriched gene into a Cre driver that responds to a pre-existing, cell-type-specific Dre line, SIRA unlocks the vast universe of Cre-responsive floxed alleles for functional studies. Generating RSR-CreER alleles for every candidate gene is a Herculean task—beyond the capacity of any single laboratory or nation. However, it should not deter us from starting. I propose a community-driven strategy: (i) build and share a public resource of major cell type-specific Dre drivers. Approximately 200 major cell types exist, a realistic goal within a few years for labs already experienced in Dre resource development (Han et al, 2021); (ii) for each newly discovered subpopulation, generate RSR-CreER alleles for the top two to three most compelling candidate genes emerging from scRNA-seq analyses. This targeted approach allows any laboratory to move rapidly from gene lists to gain- and loss-of-function experiments in vivo.

Progress will require collaboration between computational biologists, who nominate high-value markers, and geneticists, who engineer transgenic tools. Funding agencies can accelerate the transition from atlas to mechanism by supporting not only atlas-building consortia but also focused pipelines for high-priority RSR-CreER lines. Emerging technologies, such as in situ CRISPR screening (Jaitin et al, 2016) and single-cell perturbation sequencing (Dixit et al, 2016), will complement SIRA by enabling high-throughput hypothesis generation, but they cannot replace precise, cell subpopulation-specific manipulations in vivo. A key advantage of SIRA is immediate compatibility with existing Cre-loxP infrastructure: a vast number of floxed alleles are already available from repositories and commercial providers such as The Jackson Laboratory, GemPharmatech, Cyagen, and Shanghai Model Organism Corporation. Thus, each new RSR-CreER line, when paired with a relevant Dre driver, opens an actionable window into the biology of a defined subpopulation to deliver a rapid return on investment. It may become feasible to assemble a comprehensive, openly available library of gene-derived RSR-CreER alleles, analogous to today’s floxed-allele collections.

## Practical considerations and limitations

SIRA introduces greater costs, breeding complexity, and animal use than conventional Cre-loxP strategies, which may limit its accessibility for laboratories with constrained funding or those under pressure to reduce animal use. One potential solution would be community-level centralization of multiple recombinase alleles, allowing investigators to obtain pre-established lines from repositories rather than generating them independently. Successful implementation of SIRA also depends on starting with a validated cell-type-specific Dre driver, such as *Col1a2-Dre* for fibroblasts, *Cdh5-Dre* for endothelial cells, or *Alb-Dre* for hepatocytes, to ensure that *CreER* expression is first restricted to the correct lineage before engaging a subpopulation-specific gene. For RSR-CreER generation, it is advisable to prioritize 2-3 candidate genes from the differentially expressed gene list, selecting those with the highest enrichment and specificity within the same major cell type to maximize the likelihood of success. An additional challenge is that scRNA-seq typically identifies quantitative differences in transcript abundance, whereas SIRA operates as a binary switch. This discrepancy can be mitigated by selecting markers with >10-fold expression differences, optimizing tamoxifen dosage and timing to favor recombination in cells with high target gene expression, and generating *RSR-CreER* alleles for multiple top-ranked candidate genes to empirically determine the most effective line.

Compatibility among recombinase systems is another important consideration. While *Cre-loxP* and *Dre-rox* have been reported to exhibit some crosstalk (Madisen et al, 2015), *Cre-loxP* and *Flp-frt* do not, and many Flp driver lines are already available. To increase flexibility, an allele such as *Gene C-frt-Stop-frt-rox-Stop-rox-CreER* can be adapted to *Gene C-frt-Stop-frt-CreER* using *CAG-Dre*, or to *Gene C-rox-Stop-rox-CreER* using *CAG-Flp*, enabling the same Cre allele to be paired with either Dre or Flp drivers. For even greater precision, a triple-intersection strategy could be used in which *Gene A-Flp* and *Gene B-Dre* provide the first two layers of specificity, while *Gene C-frt-Stop-frt-rox-Stop-rox-CreER* restricts *CreER* activation exclusively to A⁺B⁺C⁺ cells.

Another limitation is the reduced efficiency associated with dual recombination. This may be improved using constitutively active rather than inducible Dre in the first recombination step, incorporating WPRE into the *RSR-CreER* construct to enhance translation, and testing multiple candidate marker lines to identify the most efficient configuration. Finally, SIRA can also be adapted to capture transient cellular states. In such cases, Dre can be used to drive constitutively active Cre, so that once a cell activates the state-specific marker, that event can be permanently recorded through Cre-mediated recombination. This enables lineage tracing and targeting of all cells that have passed through that transient state, even if marker expression is no longer maintained.

## Future perspectives: from mechanism to precision medicine

Looking ahead, SIRA’s impact extends beyond basic discovery. Many diseases, including fibrosis, atherosclerosis, cancer, neurodegenerative diseases, and chronic inflammation, involve pathological activation of discrete cellular subpopulations that evade traditional lineage markers. The ability to genetically access, ablate, or reprogram these subpopulations enables a new generation of precision strategies. One can envision workflows in which a patient’s single-cell transcriptome reveals a disease-driving subpopulation, and a matched SIRA-based mouse model is rapidly assembled to functionally test candidate interventions targeting that state. This is the natural convergence of discovery-driven omics with hypothesis-driven genetics. SIRA thus illuminates a path from catalogs to causality, and from mechanism to medicine, by rendering subpopulation-specific interventions experimentally tractable. What is needed now is collective will: to build, validate, and openly share SIRA resources. The “valley of death” between discovery and mechanism need not persist; with SIRA, the community can construct a durable bridge to precision therapeutics.

## Supplementary information


Peer Review File

