# Author Correction: From cell atlases to mechanisms: bridging scRNA-seq discovery with in vivo genetics

**DOI:** 10.1038/s44318-026-00872-3

**Published:** 2026-08-04

**Authors:** Bin Zhou

**Affiliations:** 1 CAS CEMCS-CUHK Joint Laboratory, New Cornerstone Science Laboratory, State Key Laboratory of Cell Biology, Shanghai Institute of Biochemistry and Cell Biology, Center for Excellence in Molecular Cell Science, Chinese Academy of Sciences, Shanghai, China; 2 CAS CEMCS-CUHK Joint Laboratory, Department of Chemical Pathology, The Chinese University of Hong Kong, Prince of Wales Hospital, Hong Kong, China; 3 Key Laboratory of Systems Health Science of Zhejiang Province, School of Life Science, Hangzhou Institute for Advanced Study, University of Chinese Academy of Sciences, Hangzhou, China; 4 School of Life Science and Technology, ShanghaiTech University, Shanghai, China

## Abstract

**Figure:**
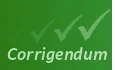

**Correction to:** *The EMBO Journal* (2026). https://doi.org/10.1038/s44318-026-00846-5 | Published online 03 July 2026

**Figure 2 corrected**

**Figure:**
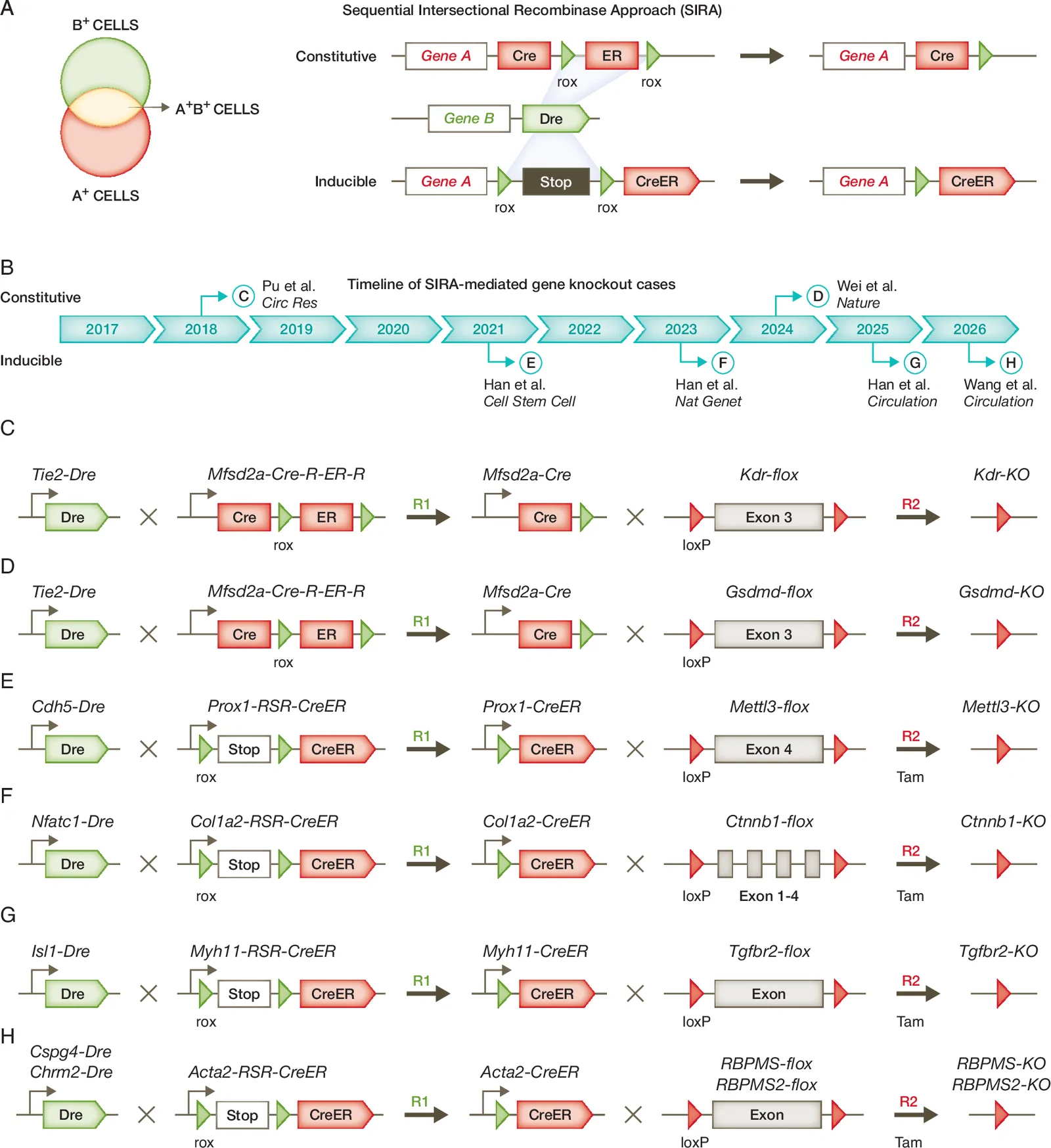

An error was identified in Figure 2 after publication and has been corrected.

The error does not affect the arguments or conclusions presented in the commentary.

