# Supplementary material for: From cell atlases to mechanisms: bridging scRNA-seq discovery with in vivo genetics
Source: EMBO J. 2026 Jul 3;45(17):5981–7. doi: 10.1038/s44318-026-00846-5 (PMC13534738; doi:10.1038/s44318-026-00846-5)
Supplement: Supplementary file 1 [file 44318_2026_846_MOESM1_ESM.pdf]

THE EMBO JOURNAL

From cell atlases to mechanisms: bridging scRNA-seq discovery with in vivo genetics

Bin Zhou

Corresponding author: Bin Zhou (zhoubin@sibs.ac.cn)

Review Timeline:

Submission Date:	24th Apr 26
Editorial Decision:	21st May 26
Revision Received:	22nd May 26
Editorial Decision:	16th Jun 26
Revision Received:	17th Jun 26
Accepted:	17th Jun 26

Editor: Daniel Klimmeck

Transaction Report:

(Note: With the exception of the correction of typographical or spelling errors that could be a source of ambiguity, letters and reports are not edited. Depending on transfer agreements, referee reports obtained elsewhere may or may not be included in this compilation. Referee reports are anonymous unless the Referee chooses to sign their reports.)

Dear Bin,

Thank you again for sending us your commentary article, as well as for your patience with our feedback. As mentioned, we have asked two dedicated experts to assess your manuscript, and in the meantime got feedback from both, which I enclose below.

As you will see, the colleagues much appreciate the perspective and initiative taken and find it highly valuable and worth publishing. They also provide constructive feedback on how to further improve it by amending technical aspects, complementing the discussion and introducing potential caveats.

I hope you will find the comments helpful. I am sure that a next version incorporating the suggestions made by the referees will be highly noted and appreciated. I would thus like to invite you to submit such a revised version using the link enclosed below.

With respect to the artwork, since no explicit comments were made by the experts, I would already pass it on to our scientific illustration team for final rendering. Please let me know, also in case I can be of any help with the text revision along the way...

Best wishes,

Daniel

Daniel Klimmeck, PhD
Senior Editor
The EMBO Journal

Referee #1:

In this comment, the author has correctly pointed out a profound limitation of single-cell RNA sequencing: gene expression differences between two cell types may imply functional difference, but sequencing alone can never demonstrate functional difference. The author proposes SIRA, a recombinase-dependent intersectional genetic strategy in genetically modified mice that restricts CreER expression to cells defined by expression of two genes. One gene would be a well-defined marker of the cell-type of interest, while the other gene would be a subpopulation-enriched marker identified through single-cell sequencing. Through expression of CreER, the cells would be rendered accessible in vivo for a wide variety of experimental manipulations aimed at determining their function(s).
Recombinase-dependent intersectional genetics is a mature technology with proven experimental value, and SIRA seems to be a reasonable extension of the general strategy. The author's suggestions that implementation of SIRA should be collaborative, community-driven, and supported by funding agencies are particularly important. However, I think the manuscript would be improved by some attention to potential limitations of the SIRA strategy, specifically including the following concerns:
1. The author mentions in several places (lines 95-96, 178) that other recombinase pairs are possible, but if SIRA is to be community-driven and collaborative, it is important that the same recombinase pair is used consistently to generate a large catalog of available recombinase strains. The author should explain why a Dre-Cre strategy is to be preferred to Flp-Cre or other dual-recombinase strategy. Specifically, the author should address published reports of crosstalk between the Cre-loxP and Dre-rox recombinase systems and assess the current availability of cell-type-specific Flp and Dre recombinase mouse lines. Searching the Jackson Lab catalog suggests that more Flp than Dre lines are publicly available.

2. In transcriptomic analyses, the differential gene expression that defines cell subpopulations may be differences in transcription levels rather than binary on/off expression. Recombinase-based strategies like SIRA, in contrast, depend on binary expression: cells are either positive for a genetic marker (and thus express a particular recombinase) or they are negative (and do not express the recombinase). There can be wide variation in levels of recombinase expression that are sufficient for experimental

outcomes (e.g. recombination of conditional knockout alleles). Therefore, it is possible that two subpopulations could be distinguished by differential expression of a particular gene in single-cell RNA-seq, but both could be "positive" by SIRA analysis. This situation may be quite common in dynamic cell states (lines 159-182) where genes are transiently up- or down-regulated. The author should further explore this potentially significant limitation of SIRA.

Referee #2:

In this insightful and stimulating commentary, Bin Zhou addresses the need to make functional sense of cellular heterogeneity unveiled by single cell RNA sequencing. Bin Zhou points out that the description of subpopulation of cells based on gene expression tells little if anything about their functions. In case a stable subpopulation of cells has been defined by a specific expression pattern, its specific function remains enigmatic until proper functional experiments are done. Bin Zhou proposes to address the function of specific subpopulations or cellular states by SIRA (Sequential Intersectional Recombination Approach), providing several examples of the successful use of this technology. He also points out that it should be feasible to target transient stages, which only occur temporarily, e.g. during disease processes. The author calls for a joint community-driven effort to generate a public resource of major cell type-specific Dre drivers and RSR-CreER alleles, which is agreeable idea. Such a resource would be of enormous help to decipher the enigma of different subpopulations and transient cellular states, offering vast opportunities.

I have little to criticize about the text. The logic is flawless and the examples well chosen. The author explains very nicely the concept of SIRA and the potential advantages. The paper is a pleasure to read and should be easily understandable also for non-specialists. The pledge to move from descriptive discoveries to mechanisms and functional experiments is well taken and should waken colleagues, who primarily indulge into descriptive mapping without further refining cell heterogeneity.

The author may consider to touch a few potential challenges and problems.

(i) The authors emphasizes that it should be possible to target transient and plastic states, if a suitable marker can be identified. True. However, it might be a challenge to time the intervention precisely. Transient cellular states usually also do not occur simultaneously but at different time points in different cells within a given time window. Any timed intervention has to take that into account to avoid misleading results. For a protracted process, it may also be difficult to administer tamoxifen for a long time. For cell tracing experiments this might be less of an issue but again, if a transient state is acquired over a long-time window, the timing of tamoxifen administration would dictate which cells are traced.

(ii) The combination of recombination events may reduce the efficiency of gene inactivation in the targeted cell population. This was not a major problem in the examples presented by the author but might become an issue in different conditions.

(iii) The generation and use of different mouse lines is costly, which is a problem in a time with dwindling funding for research in many countries. The additional breeding necessary for SIRA will increase spending on experimental animals and will extend the time needed to complete the analysis. More animals are needed when using the SIRA approach, which is an issue considering the growing critical attitude towards animal experiments. In my personal view, the advantages of SIRA outweigh such critiques but it might be prudent to address them frankly.

(iv) In principle, it should be possible to combine more than two recombinases to further increase precision. As far as I know, this has not been attempted, either because this was not necessary or because the required costs and the long duration. Nevertheless, might be mentioned as a further perspective.

Response Letter to Reviewers

Referee #1:

In this comment, the author has correctly pointed out a profound limitation of single-cell RNA sequencing: gene expression differences between two cell types may imply functional difference, but sequencing alone can never demonstrate functional difference. The author proposes SIRA, a recombinase-dependent intersectional genetic strategy in genetically modified mice that restricts CreER expression to cells defined by expression of two genes. One gene would be a well-defined marker of the cell-type of interest, while the other gene would be a subpopulation-enriched marker identified through single-cell sequencing. Through expression of CreER, the cells would be rendered accessible in vivo for a wide variety of experimental manipulations aimed at determining their function(s).

Recombinase-dependent intersectional genetics is a mature technology with proven experimental value, and SIRA seems to be a reasonable extension of the general strategy. The author's suggestions that implementation of SIRA should be collaborative, community-driven, and supported by funding agencies are particularly important. However, I think the manuscript would be improved by some attention to potential limitations of the SIRA strategy, specifically including the following concerns:

1. The author mentions in several places (lines 95-96, 178) that other recombinase pairs are possible, but if SIRA is to be community-driven and collaborative, it is important that the same recombinase pair is used consistently to generate a large catalog of available recombinase strains. The author should explain why a Dre-Cre strategy is to be preferred to Flp-Cre or other dual-recombinase strategy. Specifically, the author should address published reports of crosstalk between the Cre-loxP and Dre-rox recombinase systems and assess the current availability of cell-type-specific Flp and Dre recombinase mouse lines. Searching the Jackson Lab catalog suggests that more Flp than Dre lines are publicly available.

Author's response: Thank you for raising this important point. I agree that, for a community-driven effort, the consistent use of a single recombinase pair would be highly advantageous for building a large and interoperable catalog of mouse strains. While SIRA is conceptually compatible with either Dre-Cre or Flp-Cre combinations, our current validation examples and most of the strains developed in our laboratory, are based on the Dre-Cre system. For this reason, I used Dre-Cre examples to illustrate the practical implementation of SIRA. At the same time, I fully acknowledge that the Flp-Cre system is equally valuable and accessible for SIRA applications, and I have stated this explicitly in the revised manuscript. To address the practical challenge of system compatibility and to expand the flexibility of the SIRA framework, I have also proposed a strategy that would allow both Dre and Flp to be accommodated within a single mouse line. Specifically, an allele such as Gene C-frt-Stop-frt-rox-Stop-rox-CreER could be converted to Gene C-frt-Stop-frt-CreER by crossing with a CAG-Dre deleter strain, or to Gene C-rox-Stop-rox-CreER by crossing with a CAG-Flp deleter strain. In this way, the same Cre line could be paired with either Flp or Dre drivers, thereby broadening compatibility and making better use of existing community resources.

Regarding crosstalk, I agree that the reported cross-reactivity between Cre-loxP and Dre-rox warrants careful consideration. I have now added this caveat explicitly to the revised manuscript in the "Practical considerations and limitations" section.

2. In transcriptomic analyses, the differential gene expression that defines cell subpopulations may be differences in transcription levels rather than binary on/off expression. Recombinase-based strategies like SIRA, in contrast, depend on binary expression: cells are either positive for a genetic marker (and thus express a particular recombinase) or they are negative (and do not express the recombinase). There can be wide variation in levels of recombinase expression that are sufficient for experimental outcomes (e.g. recombination of conditional knockout alleles). Therefore, it is possible that two subpopulations could be distinguished by differential expression of a particular gene in single-cell RNA-seq, but both could be "positive" by SIRA analysis. This situation may be quite common in dynamic cell states (lines 159-182) where genes are transiently up- or down-regulated. The author should further explore this potentially significant limitation of SIRA.

Author's response: Thank you for highlighting this important limitation. I agree that single-cell RNA-seq often identifies quantitative differences in transcript abundance rather than strictly binary on/off expression, whereas SIRA, like all Cre-loxP-based systems, ultimately operates as a binary genetic switch. This challenge, however, is not unique to SIRA; it also applies to conventional Cre driver lines, in which a promoter with variable activity across related cell types may still induce recombination above a threshold in unintended populations. Several strategies may help mitigate this concern. First, marker genes should ideally be selected based on the largest fold-change differences (e.g., >10-fold) between the subpopulation of interest and the most closely related neighboring populations, as such genes are more likely to approximate a practical binary distinction. Second, the use of an inducible CreER system provides an additional level of temporal control. By adjusting tamoxifen dose and the timing of tissue collection, it may be possible to favor recombination in cells with high target gene expression over those with lower expression, particularly when the marker is transiently induced during a dynamic cellular state. Third, I recommend generating Dre- or Flp-responsive CreER alleles for two or three top candidate genes from the subpopulation signature, as empirical comparison of these lines may identify the one that most accurately captures the intended population while minimizing background recombination.

I also acknowledge that transient or dynamic cell states, where genes are only briefly upregulated or downregulated during development, homeostasis, or disease, pose a particular challenge. In these settings, combining a lineage-restricted Dre driver with a state-induced RSR-CreER allele, along with carefully controlled tamoxifen administration, may help confine labeling to cells actively passing through the transient state. Nonetheless, I agree with the reviewer that this remains a significant limitation, and I have now added an explicit discussion of this caveat in the revised text under "Practical considerations and limitations" section.

Referee #2:

In this insightful and stimulating commentary, Bin Zhou addresses the need to make functional sense of cellular heterogeneity unveiled by single cell RNA sequencing. Bin Zhou points out that the description of subpopulation of cells based on gene expression tells little if anything about their functions. In case a stable subpopulation of cells has been defined by a specific expression pattern, its specific function remains enigmatic until proper functional experiments are done. Bin

Zhou proposes to address the function of specific subpopulations or cellular states by SIRA (Sequential Intersectional Recombination Approach), providing several examples of the successful use of this technology. He also points out that it should be feasible to target transient stages, which only occur temporarily, e.g. during disease processes. The author calls for a joint community-driven effort to generate a public resource of major cell type-specific Dre drivers and RSR-CreER alleles, which is agreeable idea. Such a resource would be of enormous help to decipher the enigma of different subpopulations and transient cellular states, offering vast opportunities.

I have little to criticize about the text. The logic is flawless and the examples well chosen. The author explains very nicely the concept of SIRA and the potential advantages. The paper is a pleasure to read and should be easily understandable also for non-specialists. The pledge to move from descriptive discoveries to mechanisms and functional experiments is well taken and should waken colleagues, who primarily indulge into descriptive mapping without further refining cell heterogeneity.

Author's response: Thank you very much for carefully reading the manuscript and for all the insightful suggestions.

The author may consider to touch a few potential challenges and problems.

(i) The authors emphasizes that it should be possible to target transient and plastic states, if a suitable marker can be identified. True. However, it might be a challenge to time the intervention precisely. Transient cellular states usually also do not occur simultaneously but at different time points in different cells within a given time window. Any timed intervention has to take that into account to avoid misleading results. For a protracted process, it may also be difficult to administer tamoxifen for a long time. For cell tracing experiments this might be less of an issue but again, if a transient state is acquired over a long-time window, the timing of tamoxifen administration would dictate which cells are traced.

Author's response: Thank you for raising this important practical challenge. I agree that targeting transient and plastic cellular states is technically demanding, as these states may arise asynchronously across cells within a given time window, and prolonged tamoxifen administration is not always feasible. Several strategies may help address this issue. First, tamoxifen can be administered at the specific time point when the transient state, defined by upregulation of a candidate marker gene, is expected to occur, thereby allowing labeling and genetic targeting of cells occupying that state at that moment. Second, to capture cells that pass through the transient state at different times within a broader interval without requiring continuous tamoxifen treatment, an alternative approach is to use Flp or Dre to drive expression of a constitutively active Cre, rather than CreER, within the relevant cell type. In this configuration, once a cell activates the transient-state marker, the event is permanently recorded via Cre-mediated recombination, even if expression of the marker is later downregulated. This enables lineage tracing and genetic perturbation of all cells that have passed through the transient state, despite asynchronous timing of state entry.

(ii) The combination of recombination events may reduce the efficiency of gene inactivation in the targeted cell population. This was not a major problem in the examples presented by the author but might become an issue in different conditions.

Author's response: Thank you for raising this valid technical concern. I agree that requiring two recombinase events may reduce the overall efficiency of gene inactivation in the targeted subpopulation, as each recombination step is inherently less than 100% efficient. Several strategies may help mitigate this limitation. First, using a constitutively active Dre or Flp driver, rather than an inducible version, for the first recombination step can maximize efficiency and more reliably establish lineage restriction. Second, for the CreER-dependent second step, recombination efficiency may be improved by incorporating WPRE (woodchuck hepatitis virus post-transcriptional regulatory element) into the RSR-CreER knock-in construct to enhance transcript stability and increase CreER expression. Third, generating RSR-CreER alleles for two or three top candidate marker genes increases the likelihood of identifying at least one line with sufficient recombination efficiency for the intended subpopulation. I have added these considerations to the revised manuscript in the "Practical considerations and limitations" section.

(iii) The generation and use of different mouse lines is costly, which is a problem in a time with dwindling funding for research in many countries. The additional breeding necessary for SIRA will increase spending on experimental animals and will extend the time needed to complete the analysis. More animals are needed when using the SIRA approach, which is an issue considering the growing critical attitude towards animal experiments. In my personal view, the advantages of SIRA outweigh such critiques but it might be prudent to address them frankly.

Author's response: Thank you for raising this important practical concern. I agree that the generation and use of multiple mouse lines for SIRA increases costs, breeding complexity, experimental duration, and animal use compared to conventional Cre-loxP systems. These are legitimate challenges, particularly for laboratories with limited funding and for researchers working under increasing ethical and regulatory pressure to reduce animal use. In my view, however, the scientific advantages of SIRA, particularly its ability to enable subpopulation-specific functional studies that would otherwise be technically challenging, justify these added burdens in many contexts. At the same time, several considerations may help mitigate the practical burden. First, community-driven efforts to centralize the generation and distribution of Dre driver and RSR-CreER alleles would allow investigators to obtain pre-established lines from repositories rather than creating them *de novo*. Second, future technological advances, such as *in vivo* CRISPR-based approaches or viral delivery of recombinases, may eventually enable subpopulation-specific perturbation without the need for multiple genetically modified lines or extensive breeding. I have added a discussion of these cost and animal-use considerations to the revised manuscript in the "Practical considerations and limitations" section.

(iv) In principle, it should be possible to combine more than two recombinases to further increase precision. As far as I know, this has not been attempted, either because this was not necessary or because the required costs and the long duration. Nevertheless, might be mentioned as a further perspective.

Author's response: Thank you for raising this insightful possibility. It should, in principle, be technically feasible to combine more than two recombinases to achieve even greater precision, although to my knowledge this has not yet been demonstrated, likely due to the associated costs, breeding complexity, and experimental time required. As a conceptual extension, two orthogonal recombinases could be used in parallel, for example, Gene A-Flp and Gene B-Dre, to establish the first two layers of specificity. A subsequent triple-intersection allele, such as Gene C-frt-Stop-frt-rox-Stop-rox-CreER, could then restrict CreER expression exclusively to cells that co-express Gene A, Gene B, and Gene C (i.e., $A^+B^+C^+$). This strategy offers two potential advantages. First,

it enables genetic access to subpopulations defined with greater precision than is possible using one dual-recombinase system. Second, it broadens the SIRA framework beyond Dre-Cre by allowing Flp-based alleles to be incorporated as well. For example, the Gene C-frt-Stop-frt-rox-Stop-rox-CreER allele could be converted to Gene C-frt-Stop-frt-CreER through germline removal of the rox-Stop-rox cassette using a CAG-Dre deleter strain. I have added this perspective to the revised manuscript and included a schematic in the figure under the “Practical considerations and limitations” section.

1st Revision - Editorial Decision 16th Jun 2026

Dear Bin,

Thank you for your patience with the experts' additional feedback. We have now received recomments by the two colleagues, as shown below. As you will see they state that the commentary has been substantially improved by your amendments, and they now broadly support publication.

Hence, I am happy to share that your Comment is provisionally accepted for publication at the EMBO Journal.

I myself also went again through the manuscript text and suggest final minor changes and edits to the abstract (please compare attached version). In light of the final rendered Figures 1 and 2, please adjust the Figure legends and callouts in the text.
Also, we kindly ask you to consider additional formatting requests as to the list enclosed.

Please let me know any time if there are additional questions related.
I look forward to your last revision and moving ahead with formal acceptance and publication of this commentary shortly.

Best wishes,

Daniel

Daniel Klimmeck, PhD
Senior Editor | The EMBO Journal
d.klimmeck@embojournal.org

Instructions for preparing your revised manuscript:

>> please recheck and correct callouts for updated Figures 1 and 2.

>> please check Disclosure and competing interests statement now entered after References.

>> Reference Format: limit to 10 author names before et al.

>> Funding: enter the complete funding information into our online system.

When assembling figures, please refer to our figure preparation guideline in order to ensure proper formatting and readability in print as well as on screen:
https://bit.ly/EMBOPressFigurePreparationGuideline
See also figure legend guidelines: https://www.embopress.org/page/journal/14602075/authorguide#figureformat

Further information is available in our Guide For Authors: https://www.embopress.org/page/journal/14602075/authorguide

----

Referee #1:

The author has adequately addressed all of my suggestions in the current revision.

Referee #2:

The author has fully addressed all remarks, leaving nothing left to criticize. No further comments.

The authors addressed the remaining editorial issues.

2nd Revision - Editorial Decision 17th Jun 2026

Dear Bin,

Thank you for sending us the updated final version of the commentary article.

I am pleased to inform you that your manuscript has been accepted for publication in the EMBO Journal.

Your manuscript will be processed for publication by EMBO Press. It will be copy edited and you will receive page proofs prior to publication. Please note that you will be contacted by Springer Nature Author Services to complete licensing and payment information. Please note that as this is invited front-half content, OA charges applicable to this article will be covered.

We will proceed with transfer of the manuscript files to production shortly.

If you have any questions, please do not hesitate to contact me.

Thank you again for your kind contribution to The EMBO Journal series, which is much appreciated.

with
Best regards,

Daniel

Daniel Klimmeck, PhD
Senior Editor
The EMBO Journal

*****
Please note that it is The EMBO Journal policy for the transcript of the editorial process (containing referee reports and your response letters) to be published as an online supplement to each paper. If you should prefer removal of any referee-only figures included in the point-by-point response(s), e.g. because they may still be used for future publication or because they have been reproduced from published work by others, please do let us know immediately via response email.
More information is available here: https://link.springer.com/partners/embo-press/editorial-policies#Peer%20review

** Click here to be directed to your login page: https://emboj.msubmit.net
